# Nutraceutical approach for the management of cardiovascular risk – a combination containing the probiotic *Bifidobacterium longum* BB536 and red yeast rice extract: results from a randomized, double-blind, placebo-controlled study

**DOI:** 10.1186/s12937-019-0438-2

**Published:** 2019-02-22

**Authors:** Massimiliano Ruscica, Chiara Pavanello, Sara Gandini, Chiara Macchi, Margherita Botta, Daria Dall’Orto, Marina Del Puppo, Marco Bertolotti, Raffaella Bosisio, Giuliana Mombelli, Cesare R. Sirtori, Laura Calabresi, Paolo Magni

**Affiliations:** 1 Dipartimento di Scienze Farmacologiche e Biomolecolari, Università degli Studi di Milano, Via Balzaretti 9, 20133 Milan, Italy; 2 Dipartimento di Scienze Farmacologiche e Biomolecolari, Centro E. Grossi Paoletti, Università degli Studi di Milano, Milan, Italy; 3 Division of Epidemiology and Biostatistics, European Institute of Oncology, Milan, Italy; 4 Dipartimento di Medicina e Chirurgia, Università degli Studi di Milano Bicocca, Monza, Italy; 5 Dipartimento di Scienze Biomediche, Metaboliche e Neuroscienze, Università degli Studi di Modena e Reggio Emilia, Modena, Italy; 6 Centro Dislipidemie, ASST Grande Ospedale Metropolitano Niguarda, Milan, Italy; 7 IRCCS MultiMedica, Sesto S. Giovanni, Milan, Italy

**Keywords:** Cardiovascular risk, Probiotic, Nutraceutical, Hypercholesterolemia, LDL-cholesterol, Non-HDL cholesterol, Monacolin K

## Abstract

**Background:**

Probiotics incorporated into dairy products have been shown to reduce total (TC) and LDL cholesterolemia (LDL-C) in subjects with moderate hypercholesterolemia. More specifically, probiotics with high biliary salt hydrolase activity, e.g. *Bifidobacterium longum* BB536, may decrease TC and LDL-C by lowering intestinal cholesterol reabsorption and, combined with other nutraceuticals, may be useful to manage hypercholesterolemia in subjects with low cardiovascular (CV) risk. This study was conducted to evaluate the efficacy and safety of a nutraceutical combination containing *Bifidobacterium longum* BB536, red yeast rice (RYR) extract (10 mg/day monacolin K), niacin, coenzyme Q10 (Lactoflorene Colesterolo®). The end-points were changes of lipid CV risk markers (LDL-C, TC, non-HDL-cholesterol (HDL-C), triglycerides (TG), apolipoprotein B (ApoB), HDL-C, apolipoprotein AI (ApoAI), lipoprotein(a) (Lp(a), proprotein convertase subtilisin/kexin type 9 (PCSK9)), and of markers of cholesterol synthesis/absorption.

**Methods:**

A 12-week randomized, parallel, double-blind, placebo-controlled study. Thirty-three subjects (18–70 years) in primary CV prevention and low CV risk (SCORE: 0–1% in 24 and 2–4% in 9 subjects; LDL-C: 130–200 mg/dL) were randomly allocated to either nutraceutical (*N* = 16) or placebo (*N* = 17).

**Results:**

Twelve-week treatment with the nutraceutical combination, compared to placebo, significantly reduced TC (− 16.7%), LDL-C (− 25.7%), non-HDL-C (− 24%) (all *p* < 0.0001), apoB (− 17%, *p* = 0.003). TG, HDL-C, apoAI, Lp(a), PCSK9 were unchanged. Lathosterol:TC ratio was significantly reduced by the nutraceutical combination, while campesterol:TC ratio and sitosterol:TC ratio did not change, suggesting reduction of synthesis without increased absorption of cholesterol. No adverse effects and a 97% compliance were observed.

**Conclusions:**

A 12-week treatment with a nutraceutical combination containing the probiotic *Bifidobacterium longum* BB536 and RYR extract significantly improved the atherogenic lipid profile and was well tolerated by low CV risk subjects.

**Trial registration:**

NCT02689934.

**Electronic supplementary material:**

The online version of this article (10.1186/s12937-019-0438-2) contains supplementary material, which is available to authorized users.

## Background

Atherosclerosis-related cardiovascular (CV) diseases are associated with greater disability, morbidity for concomitant severe conditions and mortality [1]. In addition to some subjects with severe hypercholesterolemia, mostly related to genetic conditions [2], the majority of subjects with low or medium CV risk actually show moderate cholesterol elevation, together with moderate rise of related biomarkers [1]. This CV risk is often underdiagnosed and undertreated, thus representing a significant burden for the individual, especially in combination with unhealthy lifestyle habits [3]. In this field, statins are an established and widely used therapeutic option and their use has led to relevant improvements in the outcome of CV diseases [4]. However, statins are also well-known to be associated with important side-effects, such as muscle symptoms of different entity [5] and, to a lower extent, de novo diabetes mellitus development [6] indicating the need for additional drug and nutraceutical treatment options.

In all these conditions, nutraceutical approach may be a reasonable option, since in several instances a moderate-intensity (multi)treatment may offer relevant advantages over the no-treatment option or the presence of inadequate adherence to a drug therapy, due for example to adverse effects [7] or even as an add-on to low dose statins in secondary prevention patients intolerant to high dose statin [8]. Several nutraceutical compounds have been evaluated both alone and in combination in the context of moderate dyslipidemia [9, 10]. Among these, the most widely tested and used are extracts of red yeast rice (RYR), berberine, phytosterols, and stanols [11]. Interestingly, comparative studies between RYR and statins observed a smaller incidence of muscular side effects with the former treatment [12].

Recent evidence indicates that alterations of gut microbiota may be involved in the pathogenesis of systemic diseases related to CV risk, including hypercholesterolemia [13], suggesting that the use of selected probiotics with specific biological activities may be proposed for these systemic conditions. Indeed, available data suggest that the intake of selected probiotics, incorporated into a food matrix like yogurt or fermented milk, may lead to a significant reduction of total cholesterol (TC) (up to − 5.4%) and low-density lipoprotein-cholesterol (LDL-C) (up to − 16%) [14–16]. More specifically, probiotic strains showing high biliary salt hydrolase (BSH) activity [17, 18], such as *Bifidobacterium longum* BB536, may contribute to lower circulating TC and LDL-C by reducing intestinal cholesterol reabsorption [19]. As such food matrices may not be very practical for a long-term use, the incorporation of these probiotics into pharmaceutical forms, also in association with other nutraceuticals, may result in better adherence and efficacy for the management of low CV risk subjects.

The main objective of the present study was the evaluation of the efficacy and safety of a nutraceutical combination containing *Bifidobacterium longum* BB536, RYR extract, niacin and coenzyme Q10, on the improvement of LDL-C level as the primary end-point, as well as of a set of clinical and experimental markers of CV risk (secondary end-points).

## Methods

### Study design and population

**Fig. 1:**
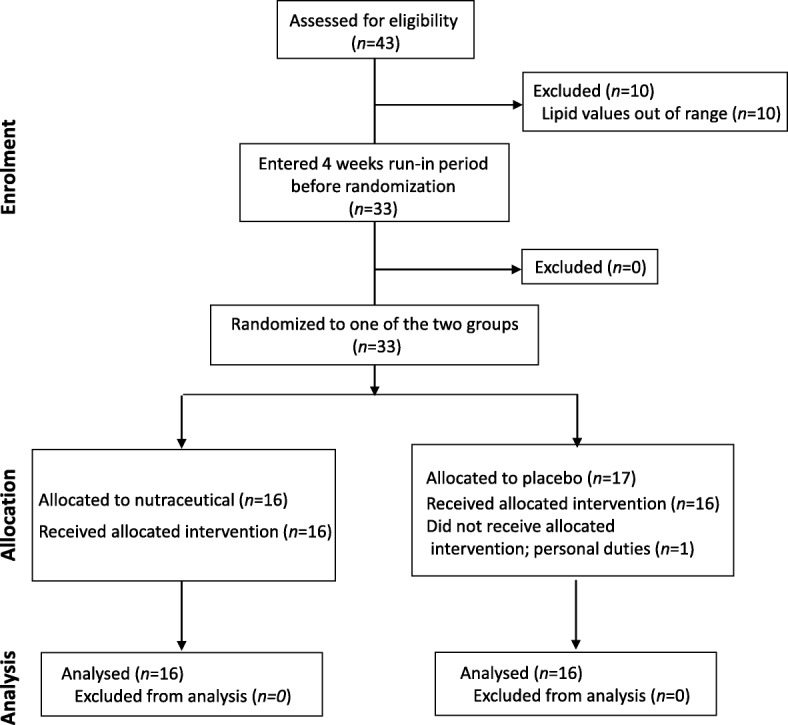
CONSORT statement flow diagram

This was a randomized, double-blind, placebo-controlled, parallel-group trial (RCT) (NCT02689934). It involved 33 subjects in primary CV prevention, with both low CV risk and LDL-C in the 130–200 mg/dL range. The study was performed at the Centro Dislipidemie (ASST Grande Ospedale Metropolitano Niguarda, Milan, Italy) in the period from November 2015 to February 2017, in accordance with the guidelines of the Declaration of Helsinki. The study was approved by the Ethics Committee of ASST Grande Ospedale Metropolitano Niguarda. A written informed consent was obtained from each subject. Sixteen males and 17 females, median aged 57 years (Q1 = 48 and Q3 = 63 years), with low total CVD risk (0–1% in 24/33 subjects (73%) and 2–4% in 9/33 subjects (27%)), as assessed by the SCORE Risk Charts (http://www.heartscore.org/en_GB/) and LDL-C levels of 180 (170, 196) mg/dL (median (Q1, Q3)) were recruited for the study (Fig. 1; CONSORT flow diagram). After a run-in period of 4 weeks, patients were randomly assigned to receive, for 12 weeks, either the nutraceutical combination - Lactoflorene Colesterolo® (1 sachet/d; granules for oral suspension) - containing 1 bn UFC *Bifidobacterium longum* BB536, RYR extract (10 mg monacolin K), 16 mg niacin, 20 mg coenzyme Q10; *n* = 16) or placebo (1 sachet/d; this latter was identical in taste and appearance to the nutraceutical combination sachet; *n* = 17) (Fig. 1). Both placebo and active treatment were packaged into a proprietary 2-compartment sachet (DUOCAM®), in order to preserve probiotic integrity. This intervention was followed by a final 4-week follow-up period.

The randomization table was obtained by computer-generated random numbers. Inclusion criteria were: subjects in primary prevention for CV disease, aged 18–70 years, LDL-C: 130–200 mg/dL, non-smokers. Exclusion criteria were: pregnancy, current or previous smoking, presence of diabetes mellitus, chronic liver disease, renal disease, or severe renal impairment treated with antidiabetic medications or insulin; untreated arterial hypertension; obesity (body mass index - BMI- ≥ 30 kg/m^2^; BMI is calculated as weight divided by height squared); any pharmacological treatments known to interfere with the study treatment; and patients enrolled in another research study in the past 90 days. To estimate the compliance, the study subjects were asked to bring back all the sachets left after the first and the second 45-day period.

### Clinical procedures

At study entry, 5 of 30 patients were on standard antihypertensive treatment, maintained for the entire duration of the study (Additional file 1: Table S1). At the screening visit, subjects were instructed to follow a normocaloric/low-saturated fat diet (Additional file 1: Table S2) and the adherence to this dietary scheme was assessed by phone calls every 2 weeks by the dietitian (RB). Clinical and biochemical evaluations were performed at the beginning and at the end of each treatment period. At all visits, patients underwent a fasting venous blood sampling and a full clinical examination, including the determination of height, body weight, waist circumference (by means of a non-stretchable tape at the umbilical level (standing position), heart rate, and arterial blood pressure. Bioelectric impedance analysis (BIA; ViScan device (Tanita Inc., Tokio, Japan)) was used to assess % abdominal fat mass (BIA (%)) and % visceral fat rating (VFR (%)), according to previously reported procedures (22). All visits were performed by the same investigator (PM), and all ViScan analyses were conducted by the same operator (RB). Plasma samples were immediately separated by centrifugation, and aliquots immediately stored at − 20 °C for subsequent assays. Primary endpoint was change in LDL-C by study arms. Secondary end points were changes in TC and other CV biomarkers (non-HDL-C, triglycerides (TG), HDL-C, apolipoprotein (apo)AI, apoB, lipoprotein(a) (Lp(a)), proprotein convertase subtilisin/kexin type 9 (PCSK9)). Data retrieval, analysis, and manuscript preparation were solely the responsibility of the authors.

### Biochemical and immunometric assays

In each blood sample, TC, TG, HDL-C, apoAI, apoB, Lp(a), fasting plasma glucose (FPG), uric acid, aspartate aminotransferase (AST), alanine aminotransferase (ALT), gamma-glutamyltranspeptidase (GGT), and creatine phosphokinase (CPK) isoenzymes were measured according to standard automated clinical procedure. LDL-C was calculated according to the Friedewald formula. Non-HDL-C was calculated as TC minus HDL-C. Commercial enzyme-linked immunosorbent assay (ELISA) kits were used according to manufacturer’s specifications and previously published protocols to quantify PCSK9 [20], fibroblast growth factor (FGF) 19 and FGF21, C-reactive protein (all from R&D System, MN). In addition, oxidized LDL (oxLDL), and insulin were also measured (Mercodia, Sweden). The homeostasis model assessment of insulin resistance (HOMA-IR) index was calculated as follows: HOMA-IR = [fasting glucose (mg/dL) * insulin (mUI/L)/405].

### Determination of serum levels of lathosterol and plant sterols

Deuterated lathosterol (0.5 μg) and 5α-cholestane (0.5 μg) were added to 0.1 mL serum samples as internal standards for the measurement of lathosterol and dietary sterols (campesterol and sitosterol), respectively. After alkaline hydrolysis with 1 mL 1 N NaOH in 90% ethanol at 60 °C for 90 min under nitrogen, samples were extracted with petroleum ether, transformed into trimethylsilyl (TMS) derivatives and analysed as described previously [21]*.*

### Gas chromatography-mass spectrometry analysis

Analysis of sterols was carried out under previously described conditions (23) monitoring ions at m/z 372 for detection of cholestane, m/z 255 and 259 for lathosterol and deuterated lathosterol, and m/z 382 and 396 for campesterol and sitosterol, respectively. Lathosterol, campesterol and sitosterol values were normalized by total cholesterol levels. Calibration curves were prepared spiking serum with fixed amounts of each internal standard and increasing amounts of the above-mentioned sterols and were treated and analysed as the samples. Concentrations were calculated on the basis of the slope of the standard curve and on the peak area ratio (sterol/internal standard) found in the sample.

### Sample size calculation

A group sample size of 16 per arm achieves 80% power to detect a difference of 20 mg/mL in absolute changes (12 weeks-0 week) in LDL-C levels (mg/mL), between the null hypothesis that in both arms the means of change in LDL-C are 10 mg/mL and the alternative hypothesis that the mean of change in LDL-C in the treatment arms is − 10 mg/mL [22]. The estimated group standard deviations were 25 mg/mL per arm, with a significance level of 5% using a two-sided two-sample t-test.

### Statistical analysis

Results are presented as median and interquartile ranges (Q1 and Q3) for all parameters. Differences in median values between treatment arms at baseline were assessed by Wilcoxon-rank sum test. The difference by treatment arms of absolute changes and percentage changes of biomarkers from baseline [12 weeks treatment - baseline (0 week)] were expressed as median and interquartile ranges. Differences in change by arms were evaluated by ANCOVA models adjusted for baseline values and uncontrolled confounding factors. Percentage changes were assessed considering 20% as cut-off point of reduction [22]. Chi-square test and multivariate logistic regression models were applied to evaluate the difference between arms in frequencies of subjects with a 20% reduction. Values of TC, HDL-C, LDL-C and TG were analyzed by repeated-measure ANCOVA models. Mixed effects models are adjusted for baseline value and include as fixed effects: time, treatment arms and age. Residual plots assessed the validity of the assumptions of the models. Least square means are obtained from the full model, adjusted for baseline values, and are presented by time and treatment arms. All tests are 2-sided, and *P* values 0.05 are considered statistically significant. Statistical analysis was performed by using the SAS Software version 9.3 (SAS, NC).

## Results

### Study population

All patients were in primary CV prevention and free from liver/kidney disorders potentially affecting the response to treatment and were not on any drug affecting lipid/lipoproteins or glycaemic profile, including thiazolidinediones or corticosteroids. The baseline clinical and biochemical data indicate that the study subjects showed low CV risk, with 73% of subjects with a SCORE risk of 0–1%. Median TC was 271 (247, 288) mg/dL and LDL-C was 180 (170, 196) mg/dL (median (Q1, Q3)) (Table 1). TG, HDL-C, body weight and BMI, waist circumference and blood pressure were within the reference range [23, 24]. Primary and secondary end points and any other clinical parameter at baseline did not differ between the nutraceutical combination group and the placebo group (Table 1).

**Table 1:** Main baseline clinical and biochemical characteristics of the study population

	Baseline values	Difference between arms at baseline (*P*-value)
Age (years)	57 (48, 63)	**0.01**
Weight (kg)	65 (62, 78)	0.43
BMI (kg/m^2^)	24 (21, 27)	0.89
WC (cm)	88 (84, 94)	0.91
BIA (%)	31 (27, 40)	0.99
VFR (%)	11 (8, 12)	0.91
SBP (mmHg)	120 (120, 130)	0.54
DBP (mmHg)	80 (80, 80)	0.88
HR (bpm)	64 (60, 68)	0.24
TC (mg/mL)	271 (247, 288)	0.51
LDL-C (mg/mL)	180 (170, 196)	0.29
HDL-C (mg/mL)	60 (43, 77)	0.51
Non-HDL-C (mg/mL)	210 (193, 228)	0.24
TG (mg/mL)	115 (94, 150)	0.79
apoAI (mg/dL)	118 (95, 133)	0.28
apoB (mg/dL)	146 (134, 155)	0.07
oxLDL (U/L)	76.6 (69.7, 86.7)	0.08
Lp(a) (mg/dL)	6 (4, 12)	0.32
PCSK9 (ng/dL)	341 (285, 403)	0.81
FPG (mg/dL)	95 (89, 98)	0.97
Insulin (mUI/L)	3.38 (2.46, 4.92)	0.64
HOMA-IR	0.75 (0.57, 1.15)	0.72
FGF19 (pg/mL)	227 (173, 337)	0.87
FGF21 (pg/mL)	179 (119, 229)	0.37

Statistically significant P-values are indicated in bold

*BMI* body mass index, *WC* waist circumference, *BIA* bioelectrical impedance analysis, *VFR* visceral fat rating, *SBP* Systolic blood pressure, *DBP* diastolic blood pressure, *HR* heart rate, *TC* total cholesterol, *LDL-C* low-density lipoprotein cholesterol, *HDL-C* high-density lipoprotein cholesterol, *TG* triglycerides, *apoA-I* apolipoprotein A-I, *apoB* apolipoprotein B, *oxLDL* oxidize LDL, *Lp(a)* lipoprotein (a), PCSK9 proprotein convertase subtilisin/kexin type 9, *FPG* fasting plasma glucose, *sICAM-1* soluble intercellular adhesion molecular 1, *HOMA-IR* Homeostatic Model Assessment of Insulin Resistance, *FGF* fibroblast growth factor

### Effect of nutraceutical treatment on biomarkers of CV risk

After 12 weeks, in the nutraceutical combination group, compared to placebo, we observed significant changes of the main atherogenic lipid parameters. LDL-C was reduced by 45 mg/dL (*p* < 0.0001), corresponding to a − 25.7% reduction, TC decreased by 45 mg/dL (*p* < 0.0001), a − 16.7%, apoB by 27 mg/dL (*p* = 0.003), corresponding to a − 17% decrease, and non-HDL-C by 45 mg/dL (*p* < 0.0001), a − 24% decrease (Table 2). Similar changes were already achieved after 6 weeks of treatment and returned to baseline values after withdrawing the nutraceutical treatment for 4 weeks (data not shown). At the end of the treatment period, circulating oxLDL levels were slightly but significantly reduced by the nutraceutical combination. ApoAI, HDL-C, TG, Lp(a), PCSK9 plasma levels were unchanged in both treatment groups (Table 2). In the placebo group, no significant variations in the circulating biochemical parameters were found. Body weight, BMI, waist circumference, systolic and diastolic blood pressure and heart rate were not affected in any of the two arms (Table 2).

**Table 2:** Summary of primary and secondary end points

	Placebo		Nutraceutical		Difference of changes between arms
	Baseline	12 weeks	Baseline	12 weeks	*P-value*
Weight (kg)	65 (64, 79)	66 (63, 80)	63 (59, 75)	64 (58, 74)	0.57
BMI (kg/m^2^)	24 (21, 27)	24 (21, 27)	24 (22, 27)	24 (22, 27)	0.72
WC (cm)	88 (84, 94)	87 (82, 91)	86 (83, 94)	91 (82, 96)	0.74
BIA (%)	31 (26, 41)	31 (27, 40)	32 (27, 37)	30 (26, 38)	0.12
VFR (%)	10 (7, 12)	11 (8, 13)	11 (8, 12)	11 (8, 12)	0.83
SBP (mmHg)	125 (110, 130)	120 (110, 128)	120 (120, 130)	120 (115, 130)	0.95
DBP (mmHg)	80 (80, 80)	80 (70, 80)	80 (80, 80)	80 (80, 83)	0.46
HR (bpm)	65 (64, 68)	64 (60, 67)	64 (60, 68)	65 (62, 71)	0.23
TC (mg/mL)	271 (256, 289)	267 (259, 293)	271 (239, 285)	208 (201, 263)	**<.0001**
LDL-C (mg/mL)	189 (174, 198)	183 (171, 202)	177 (167, 193)	136.5 (118, 151.5)	**<.0001**
HDL-C (mg/mL)	56 (48, 65)	58.5 (50, 68)	71 (43, 81)	71 (48, 88)	0.97
Non-HDL-C (mg/mL)	215 (198, 232)	217 (199, 232)	206 (189, 214)	198 (182, 208)	**<.0001**
TG (mg/mL)	113 (94, 127)	112 (99, 148)	127 (93, 156)	106 (80, 124)	0.66
apoAI (mg/dL)	113.5 (97.5, 126.5)	110 (94.5, 120)	128 (93, 139)	128 (100, 151)	**0.24**
apoB (mg/dL)	143.5 (134, 148)	135 (133, 151)	155 (137, 158)	118 (112, 131)	**0.003**
oxLDL (U/L)	71.2 (66.7, 84.0)	76.4 (71.1, 99.5)	78.3 (74.6, 129.0)	81.0 (64.5, 114.1)	**0.014**
Lp(a) (mg/dL)	4.5 (2, 15.5)	4 (2, 17.5)	7 (4.5, 12)	9 (4, 14.5)	0.20
PCSK9 (ng/dL)	341 (294, 375)	324 (259, 360)	341 (294, 375)	346 (302, 366)	0.38
FPG (mg/dL)	95 (90, 97)	93 (87, 101)	93 (89, 101)	95 (92, 103)	0.20
Insulin (mUI/L)	3.23 (2.64, 5.24)	3.04 (2.31, 6.51)	3.38 (2.24, 4.92)	3.01 (2.38, 5.12)	0.78
HOMA-IR	0.73 (0.59, 1.23)	0.69 (0.51, 1.64)	0.77 (0.52, 1.15)	0.77 (0.49, 1.24)	0.92
FGF19 (pg/mL)	233 (166, 337)	261 (164, 305)	218 (177, 299)	221 (183, 374)	0.98
FGF21 (pg/mL)	185 (72, 227)	148 (77, 263)	179 (158, 364)	161 (66, 309)	0.11

Data are shown as median (1st quartile, 3rd quartile)

Statistically significant P-values are indicated in bold

*BMI* body mass index, *WC* waist circumference, *BIA* bioelectrical impedance analysis, *VFR* visceral fat rating, *SBP* Systolic blood pressure, *DBP* diastolic blood pressure, *HR* heart rate, *TC* total cholesterol, *LDL-C* low-density lipoprotein cholesterol, *HDL-C* high-density lipoprotein cholesterol, *TG* triglycerides, *apoA-I* apolipoprotein A-I, *apoB* apolipoprotein B, *oxLDL* oxidize LDL, *Lp(a)* lipoprotein (a), *PCSK9* proprotein convertase subtilisin/kexin type 9, *FPG* fasting plasma glucose, *sICAM-1* soluble intercellular adhesion molecular 1, *HOMA-IR* Homeostatic Model Assessment of Insulin Resistance, *FGF* fibroblast growth factor

### Effects of nutraceutical treatment on cholesterol metabolism

In order to assess whether the nutraceutical-induced reduction of LDL-C and TC was due to changes in cholesterol synthesis and/or intestinal absorption, the supposed mechanisms of action of RYR extract and *Bifidobacterium longum* BB536, respectively. The circulating levels of lathosterol, marker of cholesterol synthesis, and of the dietary plant sterols beta-sitosterol and campesterol, markers of intestinal cholesterol absorption, were thus measured. In the nutraceutical combination group, compared to the placebo group, lathosterol:TC was significantly reduced by − 24% (*p* = 0.0206), whereas campesterol:TC and beta-sitosterol:TC were unchanged (Table 3).

**Table 3:** Determination of serum levels of lathosterol and plant sterols

	Placebo		Nutraceutical		Difference of changes between arms
	Baseline	12 weeks	Baseline	12 weeks	*P-value*
Lathosterol:TC	0.78 (0.62, 0.84)	0.72 (0.6, 0.82)	0.71 (0.52, 1.01)	0.57 (0.41, 0.8)	**0.0206**
Campesterol:TC	1.45 (0.78, 1.76)	1.38 (0.94, 2.12)	1.18 (0.84, 1.79)	1.41 (1.04, 1.92)	0.37
Beta-sitosterol:TC	1.80 (1.00, 2.56)	1.67 (1.28, 2.14)	1.62 (0.94, 2.44)	1.89 (1.51, 2.54)	0.47

Data are shown as median (1st quartile, 3rd quartile), *P*-values are adjusted for age and baseline values. *TC* Total Cholesterol

Statistically significant *P*-values are indicated in bold

### Safety, tolerability and compliance

Treatment with Lactoflorene Colesterolo® was well tolerated by all participants, who did not report any significant side effects, including gastrointestinal tract or neuromuscular symptoms. No changes in liver and kidney function were observed. Moreover, we observed no changes of FPG, insulin, HOMA-IR, FGF19 and FGF21 in both treatment groups (Table 2). The study subjects showed a high (97%) compliance to both treatments.

## Discussion

This double-blind RCT conducted in subjects with low CV risk was aimed at exploring the efficacy and safety of a novel nutraceutical association, combining the *Bifidobacterium longum* BB536, a probiotic with high BSH activity (18), with a RYR extract, niacin and coenzyme Q10 (Lactoflorene Colesterolo®). This nutraceutical combination is well accepted, safe and effective in terms of significant improvement of the atherogenic lipid profile. The primary endpoint was met with a − 25.7% drop in LDL-C; significant reductions were also found for TC (− 16.7%), ApoB (− 17%) and non-HDL-C (− 24%). Since a similar reduction of the lipid atherogenic markers was already achieved after 6 weeks of treatment, this could be a practical timeframe for assessing the individual response in the clinics. The LDL-C goal recommended by the EAS guidelines (LDL-C < 115 mg/dL) [25] was reached by 4/16 subjects, whereas in 7/16 participants LDL-C was lower than 130 mg/dL, after nutraceutical treatment, although an expected variability due to individual responsiveness has been observed. The efficacy of this nutraceutical combination in terms of improvement of clinical lipid markers is comparable or even better than that of several other widely used nutraceuticals, evaluated by RCT studies [26–28].

The intake of selected probiotics incorporated into milk or milk derivatives, as mentioned before, has been found to improve the lipid profile of moderately hypercholesterolemic subjects, with a significant (− 5/18%) reduction of TC and LDL-C [15, 19], indicating that this approach is relevant and needs to be further pursued. In these studies, however, the amount of food containing the probiotics was quite high (300–350 g or mL of fermented milk or yogurt per day), making such approach less practical for long-term treatments. On the other hand, we show here that is reasonably simpler, safe and even more effective, for a long-term treatment, to propose a nutraceutical combination containing a probiotic in a specific pharmaceutical form (granules for oral suspension), packaged into a 2-compartment sachet to preserve probiotic integrity. Interestingly, the extent of LDL-C modifications with this nutraceutical combination is similar to the average reduction observed in some of the earlier statin clinical trials, reported in the CTT meta-analysis [29]. Clinical trials with statins and, in general, with lipid-modifying drugs, included very large cohorts and long-term follow-ups and enabled to appreciate the reduction of major adverse CV events. This important aspect has not yet been addressed by clinical trials with nutraceuticals and remains a relevant challenge for the future. The effects of the combination of another probiotic (*L. fermentum* ME-3) with RYR and other active components on the lipid profile has also been recently evaluated in an open-label preliminary study, leading to the observation of a significant − 18% reduction of LDL-C [30]. The use of this nutraceutical combination was associated with a slight reduction of oxLDL levels. Interestingly, the circulating levels of PCSK9, the main regulator of LDL-C, were not affected by this nutraceutical combination, possibly counteracting the known stimulatory effect of RYR alone on this protein [31, 32]. Another RCT evaluating a nutraceutical mix including RYR titrated, like in the present study, at 10 mg/day monacolin K, and the antioxidant compound hydroxytyrosol, reported a significant − 20% reduction of oxLDL [33].

The potential advantage of this nutraceutical combination is that it is supposed to activate two separate mechanisms to promote LDL-C and TC reduction: inhibition of cholesterol synthesis in the liver by RYR extract and reduction of intestinal cholesterol absorption by the *Bifidobacterium longum* BB536, through its high BSH activity. The analysis of circulating levels of sterols allowed to estimate the relative contribution of the two mechanisms to the observed LDL-C and TC reduction. As expected, a marked lathosterol:TC reduction indicates a strong inhibition of cholesterol synthesis. We found no changes of campesterol:TC and sitosterol:TC upon active treatment. While this may be viewed as a negative finding, it should be noted that treatment with statins, including lovastatin, structurally identical to monacolin K, in addition to reduce liver cholesterol synthesis, with lathosterol:cholesterol reduction, tends to increase intestinal cholesterol absorption, shown by increased campesterol:cholesterol and beta-sitosterol:cholesterol ratios [26, 34].

Although no data on specific RYR-sterols interactions are available, since monacolin K is structurally identical to lovastatin, one may predict that RYR extract may behave like a statin concerning the modulation of circulating sterol levels. Our findings therefore through an indirect mechanism suggest that *Bifidobacterium longum* BB536 may effectively act to minimize a possible monacolin K-driven increase of cholesterol absorption, as suggested by the unchanged campesterol and beta-sitosterol levels. Future studies should assess the potential usefulness of probiotics like *Bifidobacterium longum* BB536 on top of cholesterol synthesis inhibitors like statins. This nutraceutical combination appears to be safe also in terms of glucose metabolism, since no changes were observed regarding insulin resistance and FGF19 and FGF21 levels [35, 36].

The strengths of this study include some novel aspects in the field of nutraceuticals for CV risk: i) the inclusion of a probiotic with a specific biological activity (BSH) in a nutraceutical combination, ii) the evaluation of experimental markers of CV and metabolic risk, in addition to clinical biomarkers, and iii) the assessment, for the first time, of biomarkers of cholesterol synthesis and absorption in a RCT with nutraceuticals. A limitation of the study is that we could not evaluate additional treatment arms with, for example, the RYR extract or the probiotic alone. On the other end, a nutraceutical formulation may be advantageous (or disadvantageous) because of its complexity and studying each single component in a clinical trial may not be very informative.

## Conclusions

A 12-week treatment with a novel nutraceutical combination containing the probiotic *Bifidobacterium longum* BB536 and RYR extract was well tolerated by subjects with low CV risk and borderline hypercholesterolemia and resulted in a significant improvement of the proatherogenic lipid profile. The use of nutraceuticals in CV prevention, as well as in other areas related to chronic diseases like oncology, is currently expanding. Future studies may address the feasibility of “multiple probiotic-only” or “probiotic plus prebiotic” approaches to moderate hypercholesterolemia/CV risk.

## Additional file

Additional file 1: **Table S1**. Concomitant medications. **Table S2.** Average daily dietary intake. (DOCX 14 kb)

## Abbreviations

apoAI: Apolipoprotein AI
apoB: Apolipoprotein B
BMI: Body mass index
BSH: Biliary salt hydrolase
CV: Cardiovascular
DBP: Diastolic blood pressure
ELISA: Enzyme-linked immunosorbent assay
FGF: Fibroblast growth factor
FPG: Fasting plasma glucose
HDL-C: High-density lipoprotein cholesterol
HMG-CoA: Hydroxymethylglutaryl Coenzyme A
HOMA-IR: Homeostatic model assessment of insulin resistance
LDL-C: Low-density lipoprotein cholesterol
Lp(a): Lipoprotein (a)
N: Number
oxLDL: Oxidized low-density lipoprotein
PCSK9: Proprotein convertase subtilisin/kexin 9
RYR: Red yeast rice
SBP: Systolic blood pressure
TC: Total cholesterol
TG: Triglycerides
TMS: Trimethylsilyl
UFC: Unit forming colonies
WC: Waist circumference
