# Correction to: Nutraceutical approach for the management of cardiovascular risk – a combination containing the probiotic *Bifidobacterium longum* BB536 and red yeast rice extract: results from a randomized, double-blind, placebo-controlled study

**DOI:** 10.1186/s12937-019-0483-x

**Published:** 2019-09-09

**Authors:** Massimiliano Ruscica, Chiara Pavanello, Sara Gandini, Chiara Macchi, Margherita Botta, Daria Dall’Orto, Marina Del Puppo, Marco Bertolotti, Raffaella Bosisio, Giuliana Mombelli, Cesare R. Sirtori, Laura Calabresi, Paolo Magni

**Affiliations:** 1 Dipartimento di Scienze Farmacologiche e Biomolecolari, Università degli Studi di Milano, Via Balzaretti 9, 20133 Milan, Italy; 2 Dipartimento di Scienze Farmacologiche e Biomolecolari, Centro E. Grossi Paoletti, Università degli Studi di Milano, Milan, Italy; 3 Division of Epidemiology and Biostatistics, IEO, European Institute of Oncology IRCCS, Milan, Italy; 4 Dipartimento di Medicina e Chirurgia, Università degli Studi di Milano Bicocca, Monza, Italy; 5 Dipartimento di Scienze Biomediche, Metaboliche e Neuroscienze, Università degli Studi di Modena e Reggio Emilia, Modena, Italy; 6 Centro Dislipidemie, ASST Grande Ospedale Metropolitano Niguarda, Milan, Italy; 7 IRCCS MultiMedica, Sesto S. Giovanni, Milan, Italy

**Correction to: Nutr J**

**https://doi.org/10.1186/s12937-019-0438-2**

Following publication of the original article [1], the authors reported an error in the affiliation of the third author, Sara Gandini. The correct affiliation should read: Division of Epidemiology and Biostatistics, IEO, European Institute of Oncology IRCCS, Milan, Italy.

## References

[1] Ruscica M, Pavanello C, Gandini S, Macchi C, Botta M, Dall'Orto D, Del Puppo M, Bertolotti M, Bosisio R, Mombelli G, Sirtori CR, Calabresi L, Magni P (2019). Nutraceutical approach for the management of cardiovascular risk - a combination containing the probiotic *Bifidobacterium longum* BB536 and red yeast rice extract: results from a randomized, double-blind, placebo-controlled study. Nutr J.

