# Supplementary material for: Nutraceutical approach for the management of cardiovascular risk – a combination containing the probiotic Bifidobacterium longum BB536 and red yeast rice extract: results from a randomized, double-blind, placebo-controlled study
Source: Nutr J. 2019 Feb 22;18:13. doi: 10.1186/s12937-019-0438-2 (PMC6387491; doi:10.1186/s12937-019-0438-2)
Supplement: Supplementary file 1 — Table S1. Concomitant medications. Table S2. Average daily dietary intake. (DOCX 14 kb) [file 12937_2019_438_MOESM1_ESM.docx]

| Medication | Patients (%) |
| --- | --- |
| ACE-I/ARB | 9.6 |
| Beta-Blockers | 3.2 |
| Diuretics | 6.4 |
| Calcium antagonists | 9.6 |
| Other drugs | 9.6 |

**Supplementary data**

**Supplemental Table 1**. Concomitant medications

ACE-I: angiotensin-converting enzyme inhibitor

ARB: Angiotensin receptor blockers

**Supplemental Table 2**. Average daily dietary intake

**MEN**

Energy (Kcal) 1807.5 ± 171.2

Carbohydrates (g) 273.9 ± 28.4

Protein (g) 78.7 ± 8.8

Lipids (g)

total (g) 51.7 ± 4.3

saturated (g) 7.9 ± 0.8

unsaturated (g) 7.4 ± 0.7

monounsaturated (g) 31.5 ± 4.7

fiber (g) 35.2 ± 3.6

**WOMEN**

Energy (Kcal) 1500.9 ± 144.8

Carbohydrates (g) 226.1 ± 20.4

Protein (g) 67.4 ± 7.3

Lipids (g)

total (g) 31.2 ± 3.2

saturated (g) 6.6 ± 0.7

unsaturated (g) 6.2 ± 0.6

monounsaturated (g) 25.6 ± 2.9

fiber (g) 31.4 ± 4.1

Values are expressed as mean±SD

Kcal, kilocalorie; g, gram
